# The Pupil in Health and Disease

**Published:** 1888-01

**Authors:** John Chase

**Affiliations:** Denver, Colorado


### THE PUPIL IN HEALTH AND DISEASE.

BY DR. JOHN CHASE, DENVER, COLORADO.

[Read before the Arapahoe County Medical Society.]

Correct diagnosis depends on a thorough study of symptoms, and the physician who would prescribe intelligently must be able to correctly interpret the varied functions of the body in health and disease. I wish to-night to call your attention for a few moments to an organ which often serves as a guide-post to the intelligent observer, pointing him to disease of distant organs.

Too little attention has been given by the general practitioner to diseases of the eye, and too many of them are deprived of valuable aid in diagnosis by neglecting to observe the action of the eye in disease of remote organs. And now, to offset that remark, I wish to assert that most of the phenomena of the pupil which are made use of in diagnosis have been recorded and classified by the general practitioner, and to him, and not to the specialists, as yet, belongs the credit of reducing to anything like available form the clinical features of the action of the pupil. Not that the specialist has been idle, for the patient toil of the histologist and physiologist had already borne fruit, and a little light has commenced to shine on one of the dark corners of medicine. Leaving out of the present discussion the examination of the deeper structures of the eye, I wish to direct your thoughts to the action of the pupil in health and disease. Allow me to remind you that normally the pupil is a perfectly round aperture in a fibro-muscular tissue, the iris. The muscular tissue is divided into circular and radiating fibers. The former comprises the sphincter of the pupil and receives its nervous impulse from a branch of the "motor oculi." Many histologists deny the existence of the radiating fibers, but the weight of evidence is for their presence, and it is quite well established that they receive their nerve supply from the sympathetic. From this it can readily be seen that *contraction* of the pupil may follow direct stimulation of the motor oculi or paralysis of the sympathetic, and that *dilatation* of the pupil may result from stimulation of the sympathetic or paralysis of the third nerve. Contraction of the pupil also occurs, 1, when the retina or optic is stimulated; 2, when the eyes are accommodated for near vision; 3, in the early stages of poisoning by chloroform and alcohol, and in nearly all stages of poisoning by opium, arsenic and some other drugs; 4, in deep sleep; 5, after local application of myotics. *Dilatation* of pupil occurs, 1, when stimulus of light is withdrawn from the eye; 2, when the eye is adjusted for distant vision; 3, in dyspnœa, during powerful irritation of the sensory nerves, during violent exercise, during poisoning by atropine and allied drugs; 4, after local application of *mydroline*.

Two antagonistic forces work upon the pupil: One, through the motor oculi as an efferent and the optic as afferent tracts, is reflex in nature; the other, "tonic" in nature, with the cervical sympathetic as the afferent channel. These two forces are readily acted upon and interfered with by diseases at a distance from the eye, and the pupil acts as an index of the condition, but great care is necessary to correctly translate the indication. The pupils should be examined as to (1) equality in size, (2) size in ordinary light, (3) mobility, (4) shape.

To successfully apply to systemic diseases the actions of the pupil we must first exclude all pathological changes due to disease of the eye itself, remembering as well that myosis, or extreme contraction of the pupil, may be caused by various drugs, such as eserine or pilocarpine, and that mydriasis is often the result of a local application of cocaine or atropine. In iritis the pupil will often react but sluggishly, and may be tied by adhesion to the lens, either partially or completely. In another large class of cases the pupil is dilated, owing to amaurosis, and it must not be forgotten that an increase of intra-ocular tension from any cause produces dilatation of the pupil. Outside of these local causes, the pupil is influenced by the condition of the general system, and it is among these cases that is found the clinical advantage of a study of the pupil. I confess to considerable disappointment in looking over the literature of the subject to find, not only a diversity of opinion, but a direct antagonism among those who have investigated closely. At present, however, nearly all agree upon one or two propositions, which will serve as a basis for further work. Whatever depresses the cerebro-spinal system, or *excites* the ganglionic system causes mydriasis; and we find *myosis* whenever the ganglionic system is depressed or the cerebro-spinal irritated. Clinical theories, then, are quite well supported.

To mention the action of the pupil in all the general diseases of the body, where it is subject to change, and to give the reason of its action, would be but to give a pathological account of them all, and so we will content ourselves with a mere enumeration of a few of the more prominent ones. Dilatation of course occurs in paralysis of the third nerve, and this in turn may be the result of meningitis, morbid growths near the nerve, periostitis at base of the skull, or involving the sphenoidal fissure, or syphilis. *Myosis* often follows obstinate constipation or irritation of digestive tract, and mydriasis occasionally occurs in disturbance of the cerebral circulation.

Apoplexy at the base of the brain causes at first dilatation, followed in the reaction by contraction of the pupil. Basilar meningitis, on the contrary, causes, first, contraction and then dilatation. In hysteria the pupil usually contracts at the commencement of the attack, and later, dilates.

Locomoter ataxia may often be ushered in by a partial paralysis of the muscles of the eye, often only the internal muscles being affected. The most frequent variety is loss of the reflex action of the pupils, while the associated action remains. By this is meant that the pupils remain stationary when shaded and exposed to light, but contract and dilate when the accommodation is called in play. This phenomenon is known as the "Argyll Robertson pupil." This is one of the most valuable of the early symptoms of locomoter ataxia. Its weakness lies in the fact that as yet we do not know how frequently it may occur in healthy persons,or those without subsequent spinal disease. But enough,perhaps,has been said to indicate the line our study should pursue. If the action of the pupil can give us any definite intelligence of pathological changes in remote organs it is unfortunate that we can not fully read its meaning in all cases, and bring to our aid in diagnosis an ally so readily seen and interrogated.—*Denver Medical Times*.

